# Survival of implants placed with the osteotome technique: An update

**DOI:** 10.4317/medoral.17130

**Published:** 2012-05-01

**Authors:** Jose Viña-Almunia, Laura Maestre-Ferrín, Teresa Alegre-Domingo, María Peñarrocha-Diago

**Affiliations:** 1 DDS. Master in Oral Surgery and Implantology. Faculty of Medicine and Dentistry. University of Valencia; 2 Associate Professor of Oral Surgery. Professor of the Master in Oral Surgery and Implantology. Faculty of Medicine and Dentistry. University of Valencia. Valencia (Spain)

## Abstract

A literature review is made to analyze the survival of implants placed with the osteotome technique. A PubMed search was made based on the key words “osteotome AND dental implants”, corresponding to publications between 1998-2008. The inclusion criteria were: a) a minimum of 10 patients; b) a minimum follow-up of 6 months; c) implants placed using the osteotome technique with or without indirect sinus lift; and d) specification of the implant number and survival rate. Sixty-four articles were identified, of which 20 met the inclusion criteria. A total of 2006 implants were placed in 1312 patients using the osteotome technique. The duration of follow-up after prosthetic loading ranged from 6-144 months. Indirect sinus lift was carried out in all but one of the studies. The residual crest height ranged from 2.3-11.7 mm. with a mean gain in bone after sinus lift of 2.5-5.5 mm. The time from implant placement to prosthetic loading varied from 1.5-9 months. The percentage implant survival rate was 90.5-100%. The survival rate of implants placed with the osteotome technique is high and does not differ with respect to implant placement with the conventional technique.

** Key words:**Osteotomes, dental implants, indirect sinus lift.

## Introduction

Summers was the first to describe the osteotome technique to increase bone density in the dental implant bed (1,2) and perform localized maxillary sinus lift (1,3). Benign paroxysmal vertigo has been reported as a complication secondary to tapping of the osteotome with the mallet (4).

The literature offers little information on the predictability of implant placement using the osteotome technique without added sinus lift. In most clinical studies, implant placement using the osteotome technique is carried out in combination with indirect sinus lift (5,6).

A literature review is made to analyze the survival of implants placed with the osteotome technique.

## Material and Methods

A PubMed search was made based on the key words “osteotome AND dental implants”, limiting the search to human studies published in English in dental journals during the period 1998-2008. The inclusion criteria were: a) a minimum of 10 patients; b) a minimum follow-up of 6 months; c) implants placed using the osteotome technique with or without indirect sinus lift; and d) specification of the implant number and survival rate. The following data were collected from each study: year of publication, inclusion criteria, type of intervention, results obtained and follow-up.

## Results and Discussion

Sixty-four articles were identified with the key words “osteotome AND dental implants”. Of these articles, 20 met the inclusion criteria and were subjected to analysis ( Table 1).

**Table 1:**
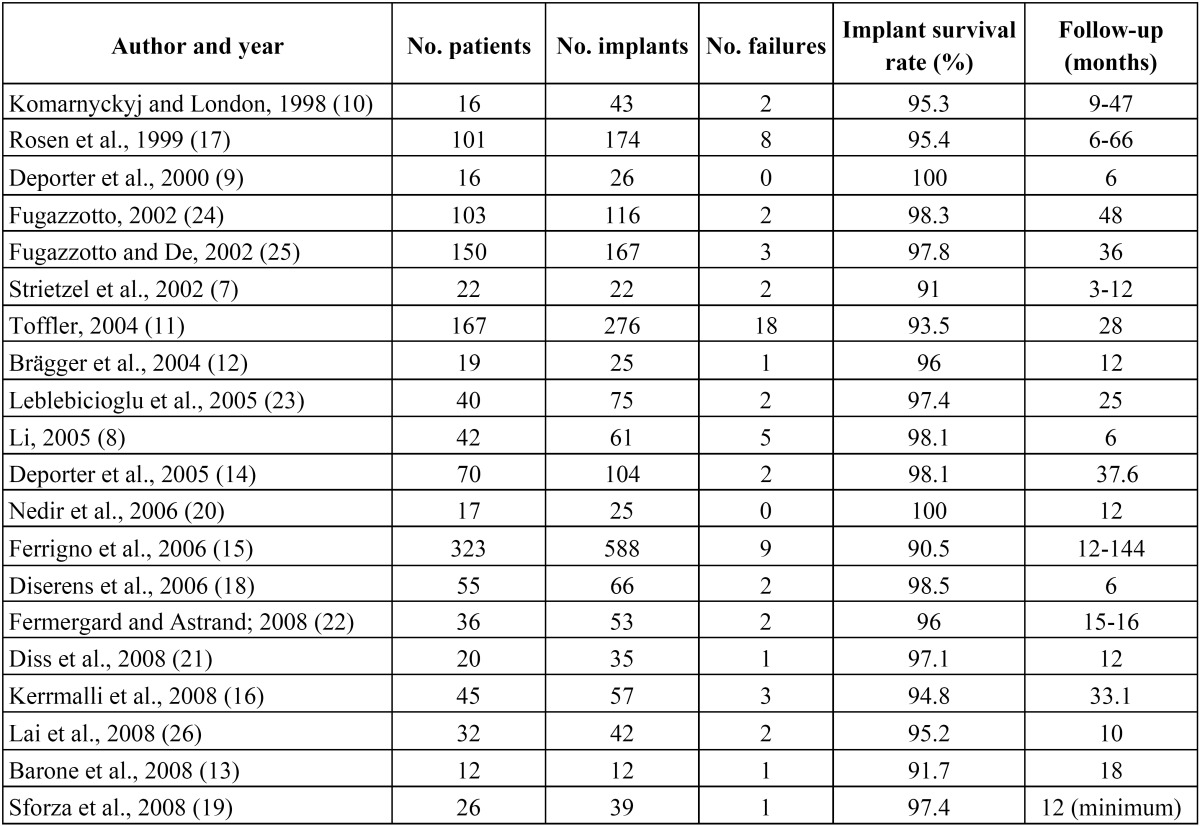
Data collected from the articles meeting the study inclusion criteria.

A total of 2006 implants were placed in 1312 patients using the osteotome technique. The duration of follow-up after prosthetic loading ranged from 6-144 months.

Indirect sinus lift was carried out in all but one of the studies (7). Specifically, Strietzel et al. used osteotomes only for alveolar crest expansion, and concluded that this technique is not indicated in Lekholm and Zarb type I and II bone, because osteotome pressure in such cortical bone adversely affects the vascular supply (7).

The residual crest height ranged from 2.3-11.7 mm in the different studies (8-16) ( Table 2). Rosen et al. (17), Diserens et al. (18) and Sforza et al. (19) performed indirect sinus lift with a minimum residual crest height of 3, 4 and 5 mm, respectively. The mean gain in bone after sinus lift was 2.5-5.5 mm (10,11,13,15,20-23) ( Table 2).

**Table 2:**
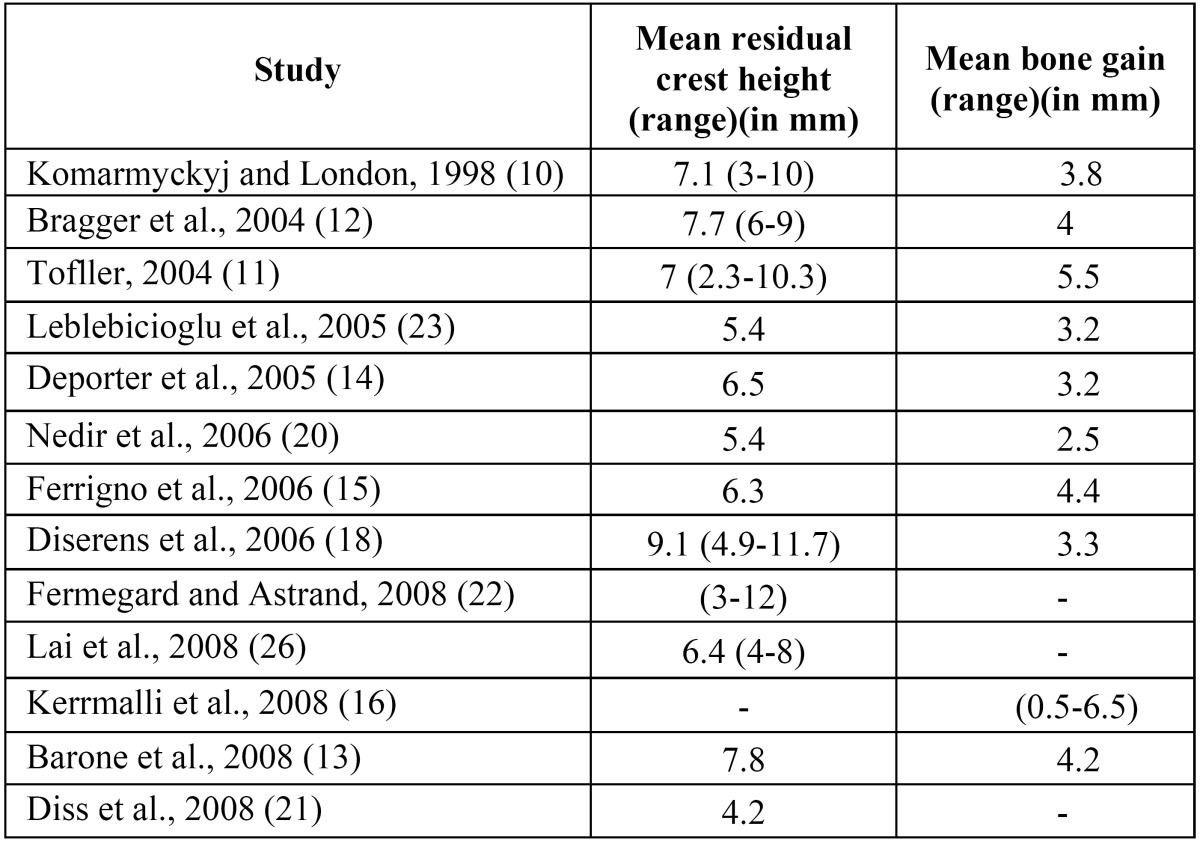
Residual crest height and bone gain in the included articles that specified these parameters.

Most of the studies used bone graft material when performing indirect sinus lift: particulate autologous bone (10,24,25), xenograft (Bio-Oss®) (9,16,18), or a combination of both (11,12,15,17,19). Five of the studies used no graft material (8,20,22,23,26). One study (21) made use of platelet-rich fibrin, while Barone et al. (13) used a mixture of collagen gel and porcine bone particles (Gel 40®, Osteobiol, Tecnoss). The implant survival rate in the sinus lift procedures made with graft material varied from 90.5-98.5%, versus 96-100% when no graft material was added.

The time from implant placement to prosthetic loading varied from 1.5 (15,21,24) to 9 months (10). In no case was immediate loading performed.

Sixty-six implants failed in 58 patients. The percentage implant survival rate with the osteotome technique was 90.5-100%. A recent study (27) observed no differences in the survival of implants placed after direct or indirect sinus lift, or in native bone in the posterior maxilla. Several authors (11,17,22) have pointed to residual bone height as a predictor of the survival of implants placed using the osteotome technique with sinus lift. Toffler et al. (11) recorded a 73.3% survival rate when the residual crest height measured 4 mm or less, versus 93.5% in the case of the total implants. Rosen et al. (17) obtained similar results: the global implant survival rate was 96% and 85.7% in the presence of residual crest heights of 4 mm or less, respectively. Fermergård et al. (22) documented two failures out of 53 implants. In both cases the residual bone height measured 4 mm or less.

## Conclusion

The survival rate of implants placed with the osteotome technique is high and does not differ with respect to implant placement with the conventional technique.
